# Computer-based fluorescence quantification: a novel approach to study nucleolar biology

**DOI:** 10.1186/1471-2121-12-25

**Published:** 2011-06-03

**Authors:** Mohamed Kodiha, Piotr Bański, Ursula Stochaj

**Affiliations:** 1 Department of Physiology, McGill University, 3655 Promenade Sir William Osler, Montreal, H3G 1Y6, Canada

## Abstract

**Background:**

Nucleoli are composed of possibly several thousand different proteins and represent the most conspicuous compartments in the nucleus; they play a crucial role in the proper execution of many cellular processes. As such, nucleoli carry out ribosome biogenesis and sequester or associate with key molecules that regulate cell cycle progression, tumorigenesis, apoptosis and the stress response. Nucleoli are dynamic compartments that are characterized by a constant flux of macromolecules. Given the complex and dynamic composition of the nucleolar proteome, it is challenging to link modifications in nucleolar composition to downstream effects.

**Results:**

In this contribution, we present quantitative immunofluorescence methods that rely on computer-based image analysis. We demonstrate the effectiveness of these techniques by monitoring the dynamic association of proteins and RNA with nucleoli under different physiological conditions. Thus, the protocols described by us were employed to study stress-dependent changes in the nucleolar concentration of endogenous and GFP-tagged proteins. Furthermore, our methods were applied to measure *de novo *RNA synthesis that is associated with nucleoli. We show that the techniques described here can be easily combined with automated high throughput screening (HTS) platforms, making it possible to obtain large data sets and analyze many of the biological processes that are located in nucleoli.

**Conclusions:**

Our protocols set the stage to analyze in a quantitative fashion the kinetics of shuttling nucleolar proteins, both at the single cell level as well as for a large number of cells. Moreover, the procedures described here are compatible with high throughput image acquisition and analysis using HTS automated platforms, thereby providing the basis to quantify nucleolar components and activities for numerous samples and experimental conditions. Together with the growing amount of information obtained for the nucleolar proteome, improvements in quantitative microscopy as they are described here can be expected to produce new insights into the complex biological functions that are orchestrated by the nucleolus.

## Background

The proper organization of cellular organelles and compartments is of fundamental importance to eukaryotic life. Within the nucleus, nucleoli are key compartments that contribute to a growing number of cellular processes [1-10]. As such, nucleoli carry out ribosome biogenesis and sequester or associate with key molecules that regulate cell cycle progression, tumorigenesis, apoptosis and the stress response [1-12]. All of these processes are directly relevant to human health and make the nucleolus a potential target for therapeutic intervention [11-14].

Defining the composition and activities of the nucleolus under various growth conditions is a prerequisite to understand its role in different aspects of cell physiology. As dynamic compartments, nucleoli are characterized by a constant flux of macromolecules, in part due to their shuttling between nucleoli and the surrounding nucleoplasm. Advanced fluorescence microscopy and photobleaching techniques revealed the highly dynamic nature of the nucleolus which is characterized by the transient association of many nucleolar proteins with this compartment [15,16]. This feature is further emphasized by the short residence time of some of the nucleolar components; for example, fibrillarin may spend less than 40 seconds in the nucleolus of BHK cells [17]. Such a dynamic organization enables the nucleolus to rapidly respond to alterations in cell physiology, coordinate the responses to these changes and communicate them to the rest of the cell [1,2,4-6]. To understand the functional organization of nucleoli, accurate, reliable and fast techniques are required which can reveal subtle differences in the molecular makeup and activities of this compartment.

Recent advances in proteomics indicate that up to several thousand different proteins could be associated with nucleoli [18-22]. However, despite the universal use of proteomic analysis and its success in identifying nucleolar proteins, it is still challenging to study the highly dynamic nucleolar proteome or the processes located in this compartment. As such, the detection of proteins that transiently associate with nucleoli is technically difficult for several reasons [21]. For instance, disruption of the cell integrity, which is necessary to isolate subcellular organelles or compartments, may lead to partial or complete disassembly of the fragile, membrane-less nucleoli. Ultimately, this may cause the loss of some nucleolar components, in particular those that are mobile and shuttle in and out of the nucleolus. On the other hand, the isolation of nucleoli from lysed nuclei by density gradient centrifugation may be complicated by cross-contamination with components that share a similar density or associate with nucleoli due to the lack of a nucleolar membrane [21]. These difficulties instigate the demand for additional techniques that can be combined with proteomic studies and generate new insights into the structural and functional organization of nucleoli. The procedures described by us provide powerful tools that serve this purpose. In particular, we present quantitative methods that rely on computer-based image analysis to measure fluorescence intensities in nucleoli. These assays employ several functions of the analysis software to first identify nucleoli and then quantify fluorescence signals that are located in this compartment. The protocols developed by us can be easily combined with automated high throughput screening (HTS) platforms, making it possible to obtain large data sets and analyze many of the biological processes that are linked to nucleoli.

## Results and Discussion

### Quantification of nucleolar fluorescence is complicated by the dynamic nature of nucleoli

**Figure 1:**
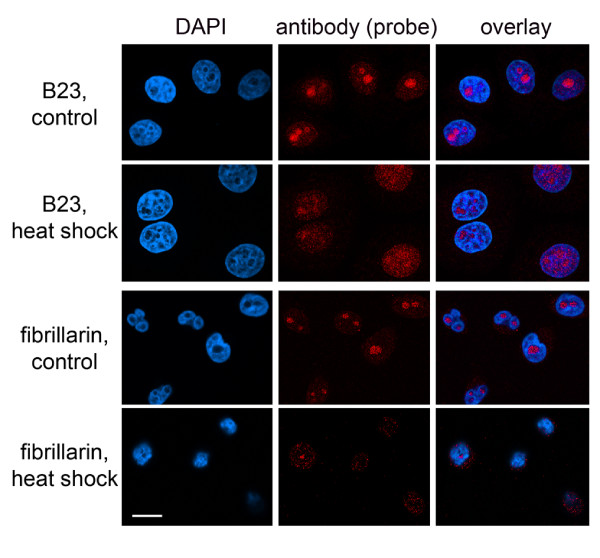
**The dynamic organization of nucleoli requires new tools for quantification of nucleolar fluorescence**. The nucleolar accumulation of B23 and fibrillarin, frequently used as marker proteins for the nucleolus, is sensitive to changes in growth conditions. HeLa cells were grown under normal conditions or exposed to a 1-hour heat shock at 45.5°C. Cells were fixed and processed for indirect immunofluorescence. Size bar is 20 μm. Note that a portion of either protein relocates from the nucleolus upon heat stress.

**Figure 2:**
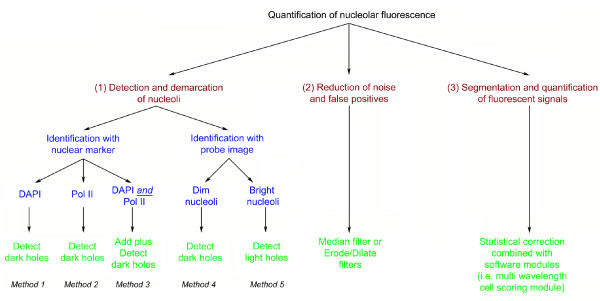
**Quantification of nucleolar fluorescence is a multi-step process**. The quantification process requires three major steps as summarized in the figure. For each of these steps images are processed with different morphology filters that are indicated in the figure. See text for details.

Measurements of organellar or compartment-specific concentrations of molecules frequently depend on a marker that identifies the subcellular structure of interest. It further requires that the marker detects a specific organelle or compartment under a variety of experimental conditions. However, in the case of nucleoli this approach is challenging, since several of the nucleolar "marker" proteins relocate in response to environmental or disease-induced changes. This is demonstrated in Figure 1 for two prominent nucleolar proteins, B23 (also known as nucleophosmin, NPM) and fibrillarin. Both proteins redistribute in response to stress, and their concentration in nucleoli can vary between different cells. Thus, B23 and fibrillarin cannot always serve as a reliable marker to define the nucleolar compartment. To overcome this obstacle, we present several distinct methods that measure pixel intensities in nucleoli (summarized in Figure 2). These procedures depend on recent advancements in computer-based image analysis and do not require a specific nucleolar marker protein. The different protocols developed by us accommodate a wide variety of experimental settings. They have been applied successfully to measure nucleolar pixel intensities for GFP-tagged reporter proteins, immunostained endogenous proteins and *de novo *synthesized RNA. These protocols measure the nucleolar fluorescence in multiple steps that involve three main operations (Figure 2): (1) Detection and demarcation of nucleoli. (2) Reduction of noise and false positives. (3) Segmentation and quantification of fluorescent signals. To perform each of these operations, different functions of the analysis software can be employed. The choice of the appropriate function is dependent on the image to be analyzed (see Methods).

To accommodate a wide diversity of experimental settings, we designed alternative methods for the analyses of images. These methods differ in the functions that are selected for each step of the quantification process. For instance, several strategies are used to identify nucleoli, and distinct functions are employed to reduce noise and false positives (Figure 2). It should be noted that some of these functions are applied to the image of the nuclear marker, while others are applied to the image of the molecule to be quantified, here referred to as probe image.

The key step in the quantification process is the identification of nucleoli. Two groups of procedures demarcate the nucleolar compartment (Figure 2). The first group (Methods 1-3) is based on a method that stains the nucleoplasm, but excludes nucleoli. The second group (Methods 4, 5) relies on the distribution of the molecule of interest which is detected with a specific probe. Methods in the second group are applicable if there is a difference in the concentration of the molecule of interest between the nucleolus and the surrounding nucleoplasm. In the following, we describe in detail each method as well as alternative protocols to analyze the distribution of nucleolar components. The basic software operations required are described in the Methods section.

### Measurement of nucleolar fluorescence based on nucleoplasmic markers with low abundance in nucleoli

In principle, these procedures rely on a nuclear marker that is absent from nucleoli or more abundant in the surrounding nucleoplasm. For example, labeling nuclear DNA with a fluorescent dye, such as 4', 6-Diamidino-2-phenylindole (DAPI), is a simple procedure to generate such a marker. Alternatively, a nuclear protein like RNA polymerase II (Pol II) can serve as a marker, because there is little Pol II in nucleoli. For both markers, the nucleoli are defined as regions within the nucleus where fluorescence intensity declines. Here, we provide three different methods that depend on the distribution of a DNA marker and/or fluorescently labeled Pol II to identify nucleoli.

#### *Method 1*. Identification of nucleoli with DAPI

**Figure 3:**
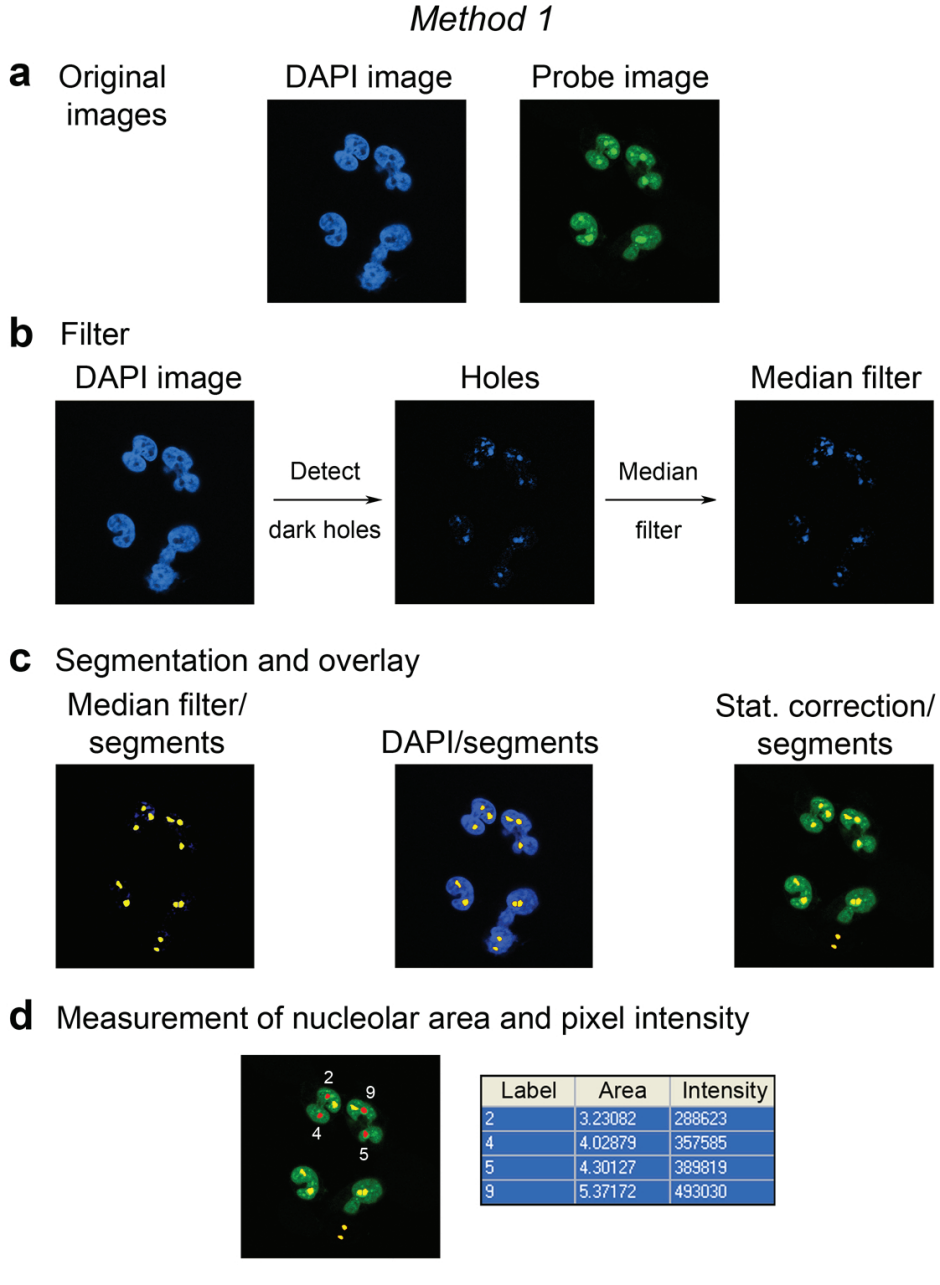
**Quantification of nucleolar fluorescence in HeLa cells synthesizing a GFP-tagged reporter protein that localizes to nucleoli and the nucleoplasm**. The DAPI image is employed to locate nucleoli. Individual operations necessary to identify nucleoli and quantify pixel intensities in the nucleolar compartment are shown. (a) Confocal images for DAPI and GFP. (b) Identification of nucleoli based on DAPI-staining of the DNA. Dark holes in the DAPI image are detected with the *Detect dark holes filter*, and noise is removed with the *Median filter*. (c) The median filter and probe images are analyzed with the *multi wavelength cell scoring *module. The median filter image serves as a compartment image to identify nucleoli, and the software generates segments which colocalize with nucleoli. The overlay of segments with DAPI and probe images verifies that nucleoli are identified correctly. (d) Areas of the nucleolar compartment in μm^2 ^and pixel intensities measured for the probe image (GFP) are depicted. Results for individual nucleoli labeled with numbers are listed in the table.

This method (Figure 3) takes advantage of the fluorescence pattern that is obtained upon incubating cells with DAPI. The procedure includes the following steps: (1) Nuclear DNA is stained with DAPI; within the brightly stained nucleoplasm nucleoli are identified as dark holes where fluorescence intensities are low [11] (Figure 3a). The molecule of interest is visualized with a probe that can be distinguished from the DAPI stain. This can be achieved with fluorescent antibodies, tags or any other suitable molecule whose fluorescence does not overlap with the DAPI signal. (2) Images are acquired by techniques that are compatible with computer-based image analyses, such as confocal microscopy or high throughput widefield image acquisition. (3) Images are analyzed with software that can be adapted to the rapid and reliable quantification of pixel intensities in the nucleolus.

In Figure 3, we applied these steps to measure nucleolar pixel intensities in transiently transfected HeLa cells that synthesized a GFP-tagged protein which is located in nucleoli and the nucleoplasm. To measure nucleolar fluorescence, cells were fixed and stained with DAPI. Images were acquired by confocal microscopy [23,24] and then analyzed with MetaXpress software (see Methods). We combined several functions of this software in an ordered fashion to delimit the nucleolar compartment. In Figure 3a, nucleoli can be discriminated from the surrounding bright nucleoplasm by the decline in DAPI fluorescence (DAPI image). Using this information, dark "holes" are identified within the nucleoplasm by applying the morphology filter *Detect dark holes*. The *Median filter *is then applied to the Holes image to reduce noise and false positives (Figure 3b, Additional file 1). As an alternative to the *Median filter*, the *Erode/dilate *function can also be used to decrease noise and false positives (see Methods).

Once the nucleolar compartments are identified, the software creates segments that represent nucleoli. The generation of segments is based on the size constraints and pixel intensity above background as they are provided by the researcher; this step is carried out for all of the methods illustrated here. The nucleolar segments are overlaid with the DAPI image (Figure 3c) and with the probe image after it has undergone statistical correction for background fluorescence (Figure 3c, Stat. correction; see Methods). After this step, fluorescence intensities are measured for the statistical correction image. The table in Figure 3d shows some of the raw data (nucleolar area and pixel intensity) that were obtained after completion of the analysis. Specifically, results for nucleoli labeled in red (2, 4, 5, 9) are displayed in Figure 3d.

#### *Method 2*. Identification of nucleoli based on Pol II distribution

Applying the same principle as described for Method 1, the distribution of Pol II, or any other marker that is of low abundance in nucleoli, can also be used to identify nucleoli (Additional file 2). This method provides an alternative solution when multiple fluorochromes are used simultaneously and their emission overlaps with the DAPI signal.

#### *Method 3*. Identification of nucleoli based on both DAPI fluorescence and Pol II distribution

**Figure 4:**
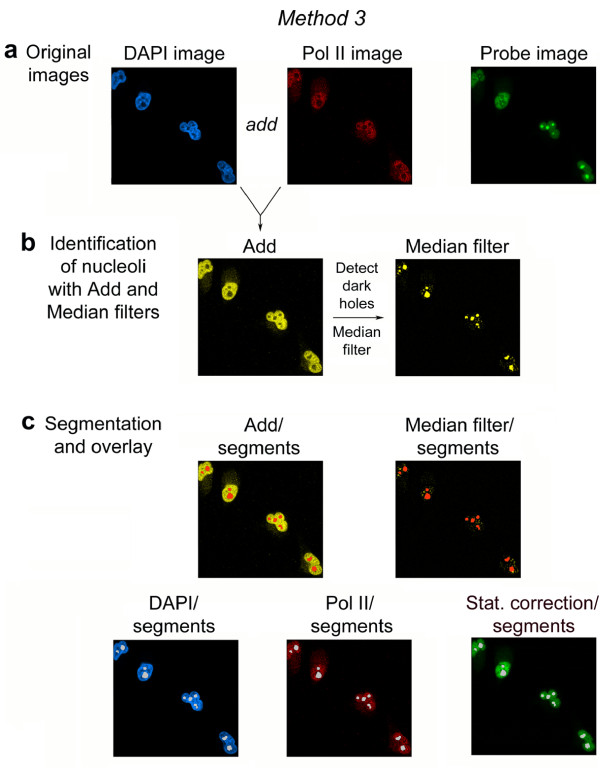
**Identification of nucleoli based on the distribution of both DAPI and Pol II**. (a) Original confocal images of HeLa cells that transiently synthesize a GFP-tagged protein. Cells are co-stained with DAPI (blue) and antibodies against Pol II (red). (b) The *Add *function is applied to DAPI and Pol II images to better demarcate nucleoli. Nucleoli are detected with the *Detect dark holes *and *Median filters*. (c) Segmentation and overlay of segments with the DAPI, Pol II and statistical correction images.

Under some experimental conditions, reliance on a single nuclear marker may not be sufficient to identify nucleoli with high confidence. Furthermore, visual inspection to exclude false positives is not always feasible, in particular when large numbers of images are generated. To address this problem, we designed an alternative method that combines information from the DAPI as well as Pol II images to identify nucleoli (Figure 4 and Additional file 2). To this end, the arithmetic *Add *function is applied to DAPI and Pol II images (Methods, Figure 4b). In the resulting image (Add image) the difference in fluorescence intensity between nucleoli and the surrounding nucleoplasm is greatly enhanced, thereby improving the subsequent detection of nucleoli by the *Detect dark holes filter*.

As shown in Additional file 2, the identification of nucleoli is more accurate for Method 3 when compared to Method 1 or 2. In additional measurements, carried out with more than 20 cells under nonstress conditions, we used DAPI combined with Pol II staining to quantify the pixel intensities for the nucleolar protein RPA194 (a subunit of RNA polymerase I). We chose RPA194 for the quantification, because it is not uniformly distributed in nucleoli and therefore particularly challenging to locate and quantify. For this experiment, the pixel intensities/area obtained for RPA194 with the *Detect light holes filter *operation was defined as 100%. Using DAPI and the *Detect dark holes filter*, 67% of the pixel intensities were measured, whereas the quantification based on Pol II and the *Detect dark holes filter *measured 74% of the fluorescence signal. The combination of the DAPI and Pol II images improved the detection of the RNA polymerase I subunit RPA194 to 80%. Taken together, Method 3 provides an improvement in the signal detection; however, one of the drawbacks of Method 3 is that it reduces the number of additional fluorochromes that can be employed in the same experiment.

### Measurement of nucleolar fluorescence based on the probe image

To accommodate different experimental settings, we developed alternative procedures (Additional file 2) that rely on the probe image to delimit nucleoli. These methods can be applied if pixel intensities in nucleoli are lower or higher than in the surrounding nucleoplasm.

#### *Method 4*. Identification of dim nucleoli with the probe image

When the fluorescence in nucleoli is dimmer than in the adjacent nucleoplasm, nucleoli can be delimited with the probe image (Additional file 2). In principle, this is the same procedure as described for Figure 3. However, the probe image, rather than the DAPI image, defines the nucleolar compartment.

#### *Method 5*. Identification of bright nucleoli with the probe image

If the fluorescence in nucleoli is brighter than in the adjacent nucleoplasm, nucleoli can be identified with the *Detect light holes filter *(Methods). Once nucleoli have been delimited, the fluorescence intensity in the defined area will be measured. We used this method to identify nucleoli based on their transcriptional activity (see below).

### The concentration of proteins B23 and fibrillarin in the nucleolus is regulated by stress

**Figure 5:**
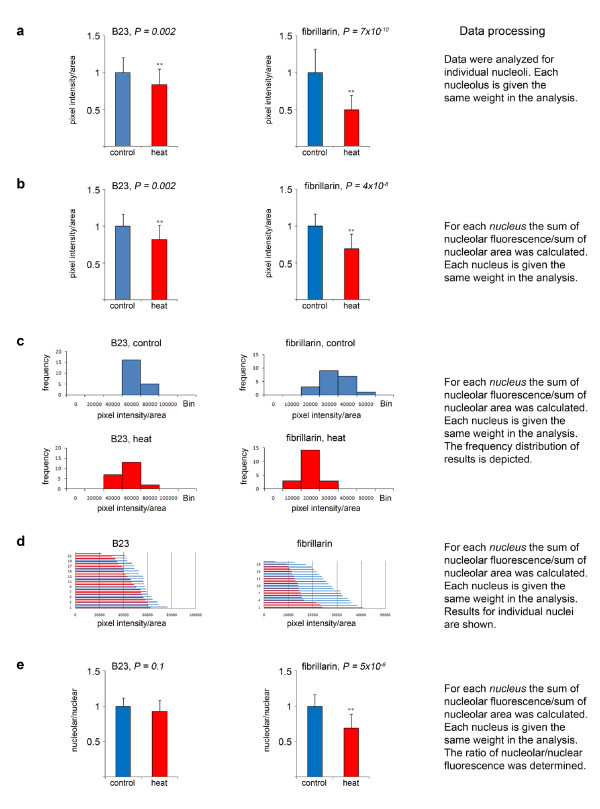
**Quantitative analysis of B23 and fibrillarin nucleolar localization**. The abundance of proteins B23 and fibrillarin in nucleoli is regulated by stress. HeLa cells were grown under normal conditions or heat-shocked for 1 hour at 45.5°C as shown in Figure 1. Nucleolar and nuclear fluorescence was quantified and pixel values/area were calculated. For B23, 21 control cells (38 nucleoli) and 22 heat-shocked cells (36 nucleoli) were measured; for fibrillarin 20 control (28 nucleoli) and 20 heat-treated cells (31 nucleoli) were analyzed. Results obtained for control conditions were defined as 1; significant differences were identified with two-tailed Student's *t-test*. Raw data were further analyzed in part a-e. (a) Fluorescence intensities were measured for all nucleoli. Individual nucleoli were given the same weight in the analysis, independent of the number of nucleoli in each nucleus. (b) Fluorescence intensities were measured for all nucleoli, average values were calculated for nucleoli if multiple nucleoli were in the same nucleus. Based on these average values, it was determined whether nucleolar fluorescence is different in control and stressed cells. (c) Changes in nucleolar fluorescence/area are shown as histograms. The same binning was applied to control and stressed samples for each protein. (d) Single cell analysis depicting nucleolar fluorescence intensities/area for individual nuclei. Results are shown for individual nuclei; each line represents the measurement for the sum of nucleoli of one nucleus of control (blue lines) or heat-shocked cells (red lines). Data were not normalized. (e) The values obtained for nucleolar fluorescence intensities in part b were used to determine the ratio of nucleolar/nuclear fluorescence intensities. Note that there is no drastic change for B23 upon heat shock, whereas a significant change is observed for fibrillarin.

We applied the approach described in Figure 3 to quantify the nucleolar concentrations of two endogenous proteins, B23 and fibrillarin, both under normal and stress conditions. To this end, control and heat-shocked cells shown in Figure 1 were used to measure pixel intensities in nucleoli. Figure 5 illustrates the information that can be obtained from the raw data acquired with our methods. In the figure we also listed details concerning the processing of raw data.

In Figure 5a, b results are displayed as nucleolar fluorescence/nucleolar area. Data were normalized to control conditions which were defined as 1. The data analysis shows that as a consequence of heat stress, the average nucleolar fluorescence decreased for both B23 and fibrillarin. Figure 5a demonstrates that the stress-induced reduction in nucleolar concentration is highly significant for B23 and fibrillarin when the analysis is performed on *individual *nucleoli.

Since many of the HeLa cell nuclei contain more than one nucleolus, we repeated the analysis after combining the measurements for individual nucleoli within the *same *nucleus into a single data point (Figure 5b). Like the analysis of individual nucleoli described above, this approach uncovers that the abundance of B23 and fibrillarin is significantly different under control and heat shock conditions. This is further confirmed when the frequency distribution of pixel intensities is examined (Figure 5c, original results for pixel intensities/area are shown). Importantly, with our approach information can be obtained about the localization of B23 and fibrillarin at the level of single cells (Figure 5d, original results for pixel intensities/area are depicted) or single nucleoli (not shown). It is noteworthy that the differences between control and heat-treated cells in Figure 5 are not suitable for a high throughput screening assay. However, the results demonstrate that small, but statistically significant, changes can be detected with our protocols under the conditions described for Figure 5.

For many proteins the balanced distribution between two different cellular compartments is critical for their biological function [24,25]. Thus, we examined whether heat stress altered the nucleolar/nuclear ratio of B23 or fibrillarin (Figure 5e). With these analyses, no drastic change in the nucleolar/nuclear ratio was detected for B23, whereas the ratio decreased significantly for fibrillarin. Since heat stress reduced the amount of B23 in nucleoli, but not the nucleolar/nuclear ratio, this may suggest that B23 relocated to the cytoplasm or was degraded upon heat shock. We tested this idea by quantifying the nuclear and cytoplasmic fluorescence in control and heat-stressed cells following published procedures [23]. This quantification showed a reduction of pixel intensities in both the nuclear and cytoplasmic compartment. However, there was no significant change in the ratio of nuclear/cytoplasmic fluorescence, which was 1 ± 0.05 (SEM, n = 26) for control and 1.13 ± 0.06 (SEM, n = 26) for heat-shocked cells. These results are consistent with the idea that the concentration of B23 decreased in response to heat stress.

Taken together, the results presented in Figure 5 reiterate the notion that a "typical" nucleolar protein may not necessarily provide an ideal marker for this compartment when physiological conditions change.

### Nucleoli can be identified with the Pol II image in heat-shocked cells

For some cell types stress may alter the DAPI staining and may therefore not be useful as the sole reference to demarcate the nucleolus. In Additional file 3 we subjected HeLa cells to heat shock and determined the distribution of Pol II by indirect immunofluorescence. We employed RPA194 as a nucleolar marker for these experiments. In previous studies we had demonstrated that the distribution of RPA194 is less sensitive to heat than B23 or fibrillarin (unpublished data). Additional file 3 shows the double staining of Pol II and RPA194 in heat-treated cells. The staining pattern obtained for Pol II was useful to define the nucleolar compartment under these conditions, and we were able to demarcate nucleoli based on Pol II alone or by the combination of DAPI and Pol II staining. Notably, although RPA194 is less affected by heat stress than other nucleolar proteins, RPA194 relocates within the nucleus in response to other treatments. Therefore, RPA194 is not suitable as a universal marker for nucleoli. By contrast, the non-nucleolar protein Pol II is useful to demarcate nucleoli under these conditions.

### Comparison of different nucleoli located in the same nucleus

Many cell types have more than one nucleolus per nucleus [11], and it is currently not understood to which extent individual nucleoli contribute to the biological processes located in this compartment. To begin to address this question, we examined whether individual nucleoli present in the same nucleus differ in the concentration of a specific protein. When pixel intensities/area were compared for individual nucleoli within the same nucleus, variability was low for B23. For the experiment depicted in Figure 5, the STDEV for nucleolar fluorescence/area within the same nucleus was 12.8% for control and 10.2% for heat-shocked cells. Variability was higher for fibrillarin (27.3% for control cells; 31.3% for heat-shocked cells; Figure 5). Future experiments will have to address the question whether the variability in fibrillarin concentration reflects functional differences between distinct nucleoli within the same nucleus.

Collectively, the analyses for B23 and fibrillarin suggest complex changes in their intranuclear location in response to stress. The studies described in Figure 5 can be easily extended to the measurement of B23 (see previous section) and fibrillarin in the cytoplasm [23,24] to provide a complete picture of their cellular distribution.

### Measurement of nucleolar fluorescence in different cell lines

**Figure 6:**
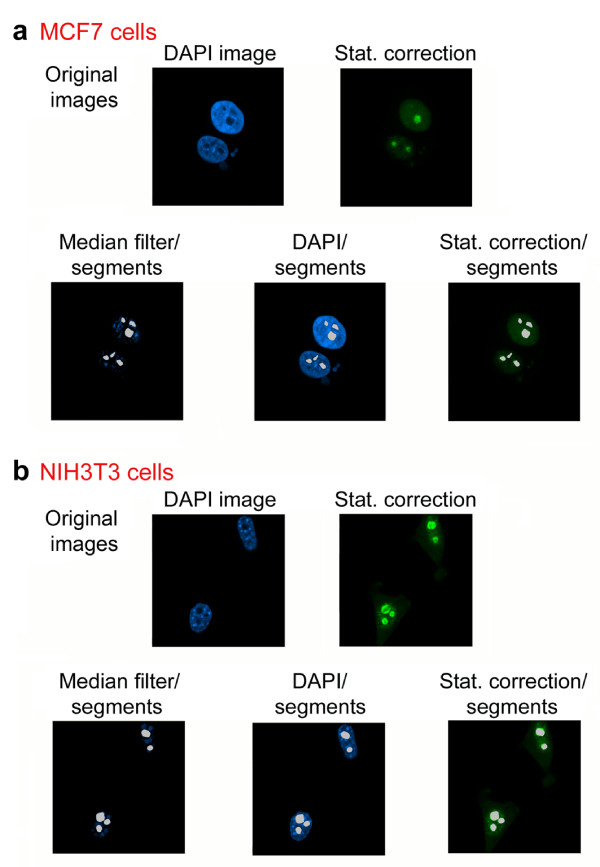
**Quantification of nucleolar fluorescence intensities in different cell lines**. Nucleoli were identified in (a) MCF7 and (b) NIH3T3 cells using the methods shown in Figure 3 for HeLa cells. Note that the unique DAPI staining pattern that is characteristic for NIH3T3 nuclei does not interfere with the quantification protocol.

Given the diversity of cellular models used in different studies, it is crucial for the methods described here to be applicable to different cell lines. This is of particular importance as nuclear morphology and the pattern obtained upon DAPI staining can differ drastically (see for instance NIH3T3 cells in Figure 6). To address this point, we quantified nucleolar pixel intensities in two different cell lines, i.e. MCF7 and NIH3T3 cells. As shown in Figure 6, the procedures initially developed for HeLa cells can be applied successfully to other cell lines.

### Analysis of the nucleolar accumulation of GFP-tagged hsc70

**Figure 7:**
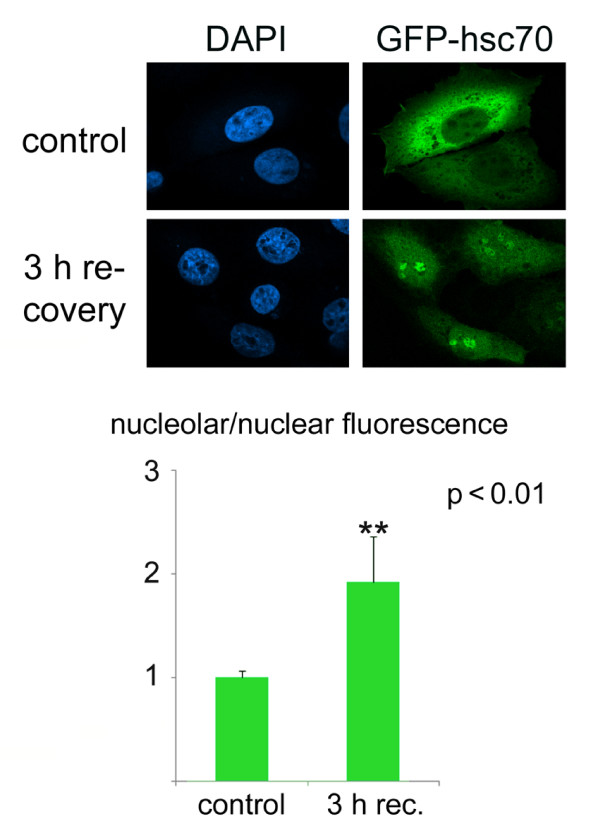
**GFP-tagged hsc70 accumulates in nucleoli during the recovery from heat stress**. Transiently transfected HeLa cells synthesizing GFP-hsc70 were grown under normal conditions or exposed to a 1-hour heat shock at 45.5°C and then incubated at 37°C for 3 hours. Nucleolar accumulation of GFP-hsc70 was measured with method 1 in controls (n = 51) and cells recovering from heat stress (n = 71). The results for nucleolar fluorescence are shown as means + STDEV; data were normalized to the control, and significant differences were determined with Student's *t-test*.

The detection of proteins by indirect immunofluorescence can be complicated by the accessibility of antigens under different conditions. Furthermore, many studies involve the analysis of reporter proteins that are fused to GFP or other fluorescent moieties. It was therefore important to demonstrate that the protocols described here can be applied to GFP-tagged proteins. In addition to the results shown in Figure 3, we monitored under different physiological conditions the distribution of GFP-hsc70, a valid model to analyze the chaperone hsc70 in growing cells [26]. Using the quantification protocols developed by us, Figure 7 demonstrates that in transiently transfected cells the nucleolar accumulation of GFP-hsc70 significantly changes during the recovery from heat stress. Since the data in Figure 7 were obtained with cells that were only transiently transfected, we did not test the suitability of this assay for high throughput screening (see below). Nevertheless, the results demonstrate that quantitative information on the changes in GFP-hsc70 nucleolar concentration can be gained with the protocols designed by us.

Taken together, the methods described here are applicable to different procedures that are used to visualize proteins or RNA (see below). This includes the labeling with antibodies, use of fluorescent reporter proteins or other techniques that generate fluorescent reporter molecules.

### Quantification of RNA synthesis in nucleoli

**Figure 8:**
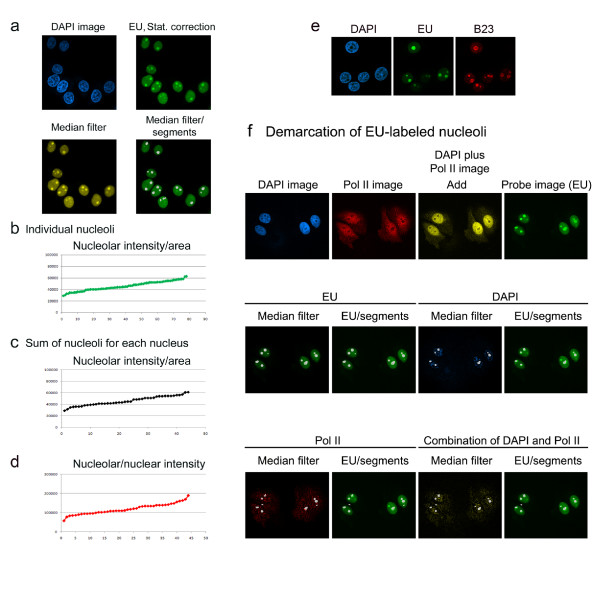
**Measurement of RNA synthesis in nucleoli**. HeLa cells were incubated with 1 mM 5-ethynyluridine for 6 hours at 37°C, fixed and treated with azide-modified Alexa Fluor488. (a) Confocal images were acquired, and the probe image was corrected for background fluorescence (EU, Stat. correction). The *Detect light holes *and *Median filters *were applied and segments were overlaid with the median filter image. (b-d) Fluorescence intensities were measured in nucleoli and nuclei of 44 cells. (b) Original data for the nucleolar intensity/area are depicted for each of the 78 nucleoli. Results were sorted smallest to largest value. The X-axis depicts individual nucleoli. (c) The sum of nucleolar pixel intensity/sum of nucleolar area is shown for each nucleus, and (d) the nucleolar/nuclear intensity was calculated for each nucleus. In (c) and (d) the X-axis displays individual nuclei. (e) HeLa cells were incubated with EU as described for part a, and B23 was subsequently located by staining with antibodies. (f) For the demarcation of EU-labeled nucleoli HeLa cells were treated with EU as described for part a, then incubated with antibodies against Pol II and Cy3-labeled secondary antibodies. DNA was stained with DAPI. Top panels show the DAPI and Pol II images; the image obtained after applying the *Add *function and the EU image. Nucleoli were then identified based on the EU image (with the *Detect light holes *and *Median filter *functions) or the DAPI image (*Detect dark holes *and *Median filter*). In the bottom panels, Pol II staining (*Detect dark holes *and *Median filter*) or a combination of DAPI and Pol II staining (*Add *function, then *Detect dark holes *and *Median filter *operations) served as references to define nucleoli.

One of the hallmarks of nucleoli is the synthesis and processing of 45S ribosomal RNA and the subsequent assembly of ribosomal subunits. Transcription can be detected in growing cells with click chemistry, and nucleoli are prominently labeled with this technique [27]. Here, we applied this method to label RNA with 5-ethynyluridine (EU) and Alexa Fluor488. Following staining with DAPI, nucleolar fluorescence was quantified by the identification of bright holes in the probe image (see above, Figure 2 and Method 5). Figure 8a displays this experiment; the quantification of pixel intensities was carried out for 44 cells. Original results for nucleolar intensity/area are depicted for individual nucleoli (Figure 8b), the sum for all nucleoli within a particular nucleus (Figure 8c) as well as the nucleolar/nuclear intensity (Figure 8d). Among individual nucleoli pixel intensities/area varied by a factor of 2 (Figure 8b), and the same variability was observed for the sum of nucleoli in each nucleus (Figure 8c). By contrast, the nucleolar/nuclear intensity showed a variability of ~3 (Figure 8d). Future experiments will have to determine whether this variability is linked to cell-cycle progression or other biological processes.

We further examined whether the staining pattern obtained for EU-labeling properly identified the nucleolar compartment. To this end, *de novo *synthesized RNA was labeled as described above, and B23 was detected by immunostaining (Figure 8e), which demonstrated the colocalization of EU with B23 in nucleoli. Moreover, our protocols can be used to identify nucleoli as they are defined by EU staining. In Figure 8f cells were labeled with EU, fixed and stained with DAPI and antibodies against Pol II. For these samples, EU-labeled nucleoli can be properly defined with the DAPI or RNA Pol II image or a combination of DAPI and RNA Pol II images. Thus, the nucleoli demarcated with our methods colocalized with the regions identified by newly synthesized RNA.

To evaluate whether EU staining can provide a marker for the nucleolar compartment upon stress, we exposed cells to a 1-hour heat shock either before or after the incubation with EU. Under these conditions, EU labeling remained prominent in the nucleolus (Additional file 4). Nevertheless, an increased number of cells displayed higher signals for EU in the nucleoplasm or showed nucleolar fragmentation.

### Different quantification methods give comparable results

**Figure 9:**
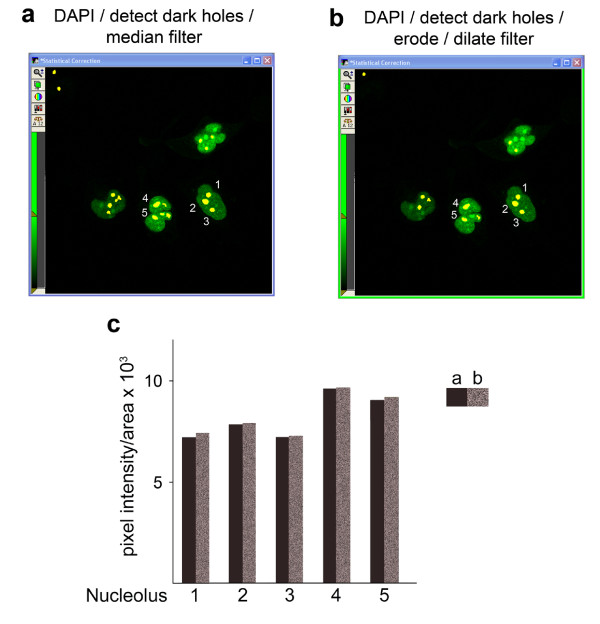
**Comparison of methods for the quantification of fluorescence associated with nucleoli**. Nucleoli were identified with the DAPI image as indicated in the figure. The different filter operations, which were performed to reduce noise and false positives, are listed on top of each panel. (a) Nucleoli were identified with the DAPI image; *Detect dark holes *and *Median filters *were applied. (b) Nucleoli were identified with the DAPI image; *Detect dark holes *and *Erode/dilate filters *were applied. (c) As shown in the graph, similar results were obtained with both protocols.

In light of the different strategies that can quantify pixel intensities in nucleoli, it was important to compare these methods. In Figure 9, the same image was used to identify nucleoli based on the DAPI staining. However, different filters were employed to demarcate the nucleolar region. The raw data are depicted for five nucleoli which are numbered in the images. Measurements of pixel intensity/area (Figure 9c) reveal that similar values are obtained with both protocols for each nucleolus, indicating that these processing operations generate comparable data.

### Automated processing of images

For the methods described here, the identification of nucleoli and measurement of nucleolar pixel intensities require several operations. These include the processing of multiple images which are generated during the analysis. As an example, Additional file 5 presents a step-by-step application of the method described in Figure 3. After completion of the operations depicted in Additional file 5a, results are obtained for the area and fluorescence intensity of individual nucleoli.

The repeated application of a large number of steps can be tedious and time-consuming. However, the work load can be reduced by automated processing of the images. Once the proper steps for image analysis have been defined (Additional file 5a), individual steps are combined into a journal (Additional file 5b). This will simplify and accelerate the processing and is particularly important for large data sets. The experiment in Figure 3 is shown here as an example; appropriate journals can be written for all the different applications discussed by us. Fluorescence signals were measured with the *multi wavelength cell scoring module*, which requires that size constraints and pixel intensity above local background are provided for the region of interest (Additional file 5c). The software then obtains data for each individual nucleolus in the probe image, and results can be saved automatically in Excel files. To confirm the proper identification of nucleoli, it is important to verify with a small number of representative cells that segments generated by the software colocalize with the "dark holes" in nuclei. If this is not the case, individual parameters of the software have to be adjusted to achieve colocalization.

### Compatibility with high throughput screening technology

**Figure 10:**
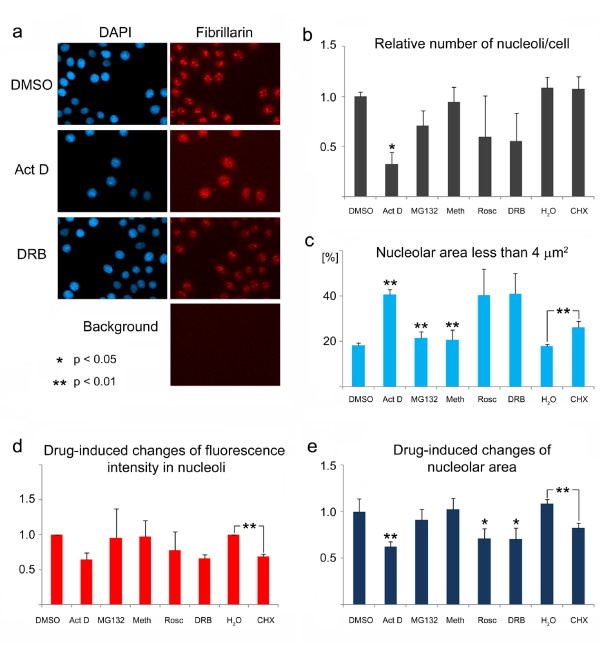
**Analysis of nucleoli with high throughput screening technology**. (a) Original images of HeLa cells stained with DAPI (blue) and anti-fibrillarin antibodies (red) were acquired with a high throughput imaging system equipped with a widefield optical imaging unit. Cells were incubated with the drugs indicated or the vehicle only (water for cycloheximide, DMSO for all other samples) and processed for indirect immunofluorescence as described in the Methods section. (b-e) Following correction for background, the probe image was analyzed for the presence of nucleoli, which were defined as bright holes that are between 2 and 5 μm^2 ^in size and contain pixel intensities larger than the user-defined threshold. The total number of cells was determined with the DAPI image. It should be noted that the background correction and data analysis were performed in a fully automated fashion without visual inspection of the images. In parts b-e results for three independent experiments are depicted as average + STDEV. (b) For each condition the total number of nucleoli was divided by the number of cells; the results were normalized to DMSO-treated samples. (c) The area stained with anti-fibrillarin antibodies was used to evaluate the effect of different compounds. The threshold was set at 4 μm^2^; and the percentage of nucleoli below this threshold was determined for each treatment. Several of the drugs increased the percentage of nucleoli that are smaller than 4 μm^2^. (d, e) The effects of drugs on the pixel intensity (d) and nucleolar area (e) were measured. Statistically significant differences for drugs delivered in DMSO were determined by One-way ANOVA. Data obtained for water and cycloheximide were compared by Student's *t-test*. Act D, actinomycin D; Meth, methotrexate; Rosc, roscovitine; DRB, 5,6-dichloro-1-beta-D-ribofuranosylbenzimidazole; CHX, cycloheximide.

High throughput screening (HTS) technology is a powerful tool to explore different aspects of cell biology, and it can be expected that research on the nucleolus will benefit from the applications of HTS technology. It was therefore important to test the suitability of our protocols for automated HTS platforms. HTS applications have to overcome the limitation that the majority of high throughput imaging systems is equipped with widefield microscopes. To address this point, we applied the "detect light holes" protocol (Method 5) to measure nucleolar fluorescence intensities for fibrillarin in images acquired with HTS technology. The protocol was applied to the probe image, thus it was not necessary to use the DAPI image as a reference. For the results depicted in Figure 10, cells were grown in 96-well plates and incubated with compounds that affect ribosome biogenesis, albeit to different degrees [28]. The compounds tested by us included actinomycin D, MG132, methotrexate, 5,6-dichloro-1-beta-D-ribofuranosylbenzimidazole (DRB), roscovitine and cycloheximide; control cells were incubated with DMSO or water. Three separate wells were incubated with every drug for each of three independent experiments. The distribution of fibrillarin was monitored for each sample, and nonspecific fluorescence was measured in a region of the well that was devoid of cells (Figure 10a).

Two to four regions were visited in each well; the probe image was corrected for background fluorescence and analyzed for "light holes". Light holes were identified as regions that are 2-5 μm^2 ^in size and show fluorescence intensities above a user-defined threshold. Each image was scored for compartments that fit these constraints. It should be emphasized that the background correction and data analyses were performed automatically on the whole set of images without visual inspection of the segmentation. However, the correctness of the segmentation was verified for several randomly chosen images.

The total number of cells was determined with the DAPI image, and the ratio of nucleoli/cell was calculated (Figure 10b). All data were normalized to the DMSO control. Note that upon treatment with actinomycin D the number of nucleoli/cell that fit the criteria for size and fluorescence intensity was significantly reduced.

To simplify the analysis, we scored changes to the nucleolar compartment by setting a threshold for the nucleolar area at 4 μm^2^. Figure 10c depicts for each of the treatments the percentage of nucleoli that are smaller than 4 μm^2^. This analysis has the advantage that it is simple and fast, without the need for extensive processing of the data; it is therefore particularly useful for high throughput applications.

A more detailed examination demonstrates the drug-induced changes to the fluorescence intensity and nucleolar area as observed with fibrillarin (Figure 10d, e). When compared to control samples, a drastic decrease in nucleolar fluorescence was observed for actinomycin D, DRB and cycloheximide (water is the control for cycloheximide, DMSO for all other drugs). In addition, a decrease in the nucleolar area was induced by actinomycin D, roscovitine, DRB and cycloheximide.

**Figure 11:**
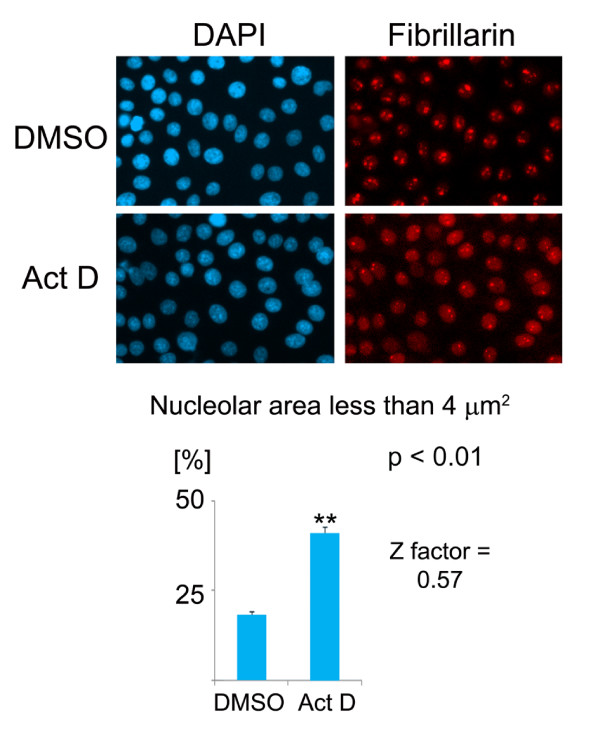
**Determination of the Z factor for HTS**. HTS conditions described in Figure 10 were used to determine the Z factor of the assay. HeLa cells were incubated with the vehicle DMSO or actinomycin D (Act D). Cells were fixed and fibrillarin was visualized by indirect immunofluorescent staining. Images were acquired and processed as for Figure 10, and the percentage of nucleoli that are smaller than 4 μm^2 ^was measured. The values for average + STDEV, obtained for three independent experiments, are depicted. On the basis of these results a Z factor of 0.57 was calculated.

We further evaluated whether our assay is robust enough to be employed in HTS experiments. Actinomycin D is a well established drug to analyze nucleoli, whereas compounds like DRB or roscovitine are known to affect nucleolar as well as other activities. Therefore, we employed actinomycin D as the reference to calculate the Z factor for our assay. Following published protocols [29], we determined the Z factor for the measurements shown in Figure 10c, which examined the percentage of nucleoli with an area smaller than 4 μm^2^. Figure 11 depicts the results for three independent experiments in which cells were treated with DMSO or actinomycin D. We applied the methods described by Zhang et al. [29] and calculated the Z-factor for our assay to be 0.57; this classifies the assay as excellent for screening.

It should be noted that with the experimental setting described for Figure 10, nucleoli cannot be demarcated with the fibrillarin staining. However, the HTS assay scored the changes in nucleolar organization based on fibrillarin distribution, thereby enabling us to quantify the drug-induced effects on the nucleolus. Taken together, our studies provide proof-of-concept that the methods described by us can be applied in a high throughput setting to quantify changes in nucleolar organization and function.

## Conclusions

Given the complex and dynamic composition of the nucleolar proteome [18-22,30,31], it is challenging to link modifications in nucleolar composition to downstream effects. One step towards this goal is the development of protocols to quantify individual components of the nucleolus in a fashion that is fast, accurate and applicable to a large number of experimental settings. Although fluorescence microscopy can be used to visualize molecules in nucleoli, this method remains rather qualitative and subtle changes in the concentration of nucleolar components can be easily missed. As an alternative method to examine the nucleolar proteome, quantitative mass spectrometry has addressed different aspects of nucleolar organization [15,30,31]. One disadvantage of this technique is the necessity to purify nucleoli, possibly leading to the loss of components that are not tightly bound. Thus, for the accurate quantification of molecules located in nucleoli, alternative strategies are needed that circumvent these limitations. To this end, we developed protocols that combine microscopy with computer-based image analysis to measure pixel intensities in nucleoli in a quantitative and reliable fashion. The methods described here keep the structural integrity of cells and nucleoli intact, they are easy to use and applicable to nucleolar molecules that are present in very low abundance or only loosely associated with this compartment. We demonstrated the effectiveness of these techniques by monitoring the dynamic association of proteins B23 and fibrillarin with nucleoli under different physiological conditions. Furthermore, we showed the usefulness of our protocols to detect proteins in nucleoli that are fused to a fluorescent reporter, and to quantify newly synthesized RNA that is associated with nucleoli. Among the protocols discussed here Method 1 is the simplest, and we applied it successfully to several different cell lines. Additional methods described by us are valuable to measure nucleolar fluorescence under more specialized conditions.

Signals that can be quantified with the presented techniques go beyond those derived from proteins. We show that our protocols can be easily applied to RNAs or biological activities that are located in nucleoli, as exemplified by the transcription of rRNA genes. Collectively, our methods provide a multi-disciplinary approach that generates information not only at the level of single cells or single nucleoli, but also for large populations of cells. One of the benefits of the protocols designed by us is their compatibility with state-of-the-art fluorescence microscopy. We provide proof-of-principle that these procedures can be combined with high throughput image acquisition using HTS automated platforms. Developing these methods sets the stage to quantify nucleolar components and activities for numerous samples and experimental conditions. Together with the growing amount of information emerging for the nucleolar proteome, methods in quantitative microscopy as they are described here can be expected to produce new insights into the complex biological functions that are orchestrated by the nucleolus.

## Methods

### Growth of cells and heat shock

HeLa S3 cells were grown on poly-L-lysine coated cover slips and exposed to heat shock as previously described [23,24,26]. Cells were fixed immediately after heat shock. For analysis with high throughput instrumentation, cells were grown and processed on 96-well plates (Costar, 96-well half area plates).

### Immunofluorescence and confocal microscopy

Fixation of cells, permeabilization and incubation with antibodies followed published procedures [23,24,26]. Antibodies against B23 were purchased from Cell Signaling Technology (#3542) and used at a dilution of 1:1,000. Antibodies against fibrillarin, Pol II and RPA194 (Santa Cruz Biotechn. sc-11335, sc-25397, sc-9001; sc-48385) were diluted 1:1,000, 1:500 or 1:200. Primary antibodies were detected with Cy3- or FITC-labeled secondary antibodies, and nuclei were stained with DAPI.

Confocal microscopy was carried out essentially as described [23,24]. In brief, confocal images were acquired with a Zeiss LSM 510 with a 63x magnification (NA = 1.4) at scan speed 5, with a pixel scanning time of 6.4 μsec. Images were acquired with 4-line averaging and a pixel resolution of <0.7 μm.

### Measurement of transcription associated with nucleoli

RNA was labeled by incorporation of 5-ethynyluridine (EU) using click chemistry as described [27]. In brief, HeLa S3 cells were grown in the presence of 1 mM EU for 6 hours according to the manufacturer's instructions (Invitrogen). Incorporated EU was labeled with Alexa Fluor488 and DNA was stained with 1 μg/ml DAPI for 2 min. Samples were mounted and images were acquired by confocal microscopy as described above.

### High throughput screening and image acquisition

For experiments shown in Figure 10 and 11, images were collected with an ImageXpress Micro equipped with a 40x objective (NA = 0.60) and a CoolSnap HQ camera. Exposure times were 20 ms for DAPI and 5 sec for TRITC. The following drugs were tested for their effect on the distribution of fibrillarin using high throughput instrumentation [23]: 100 nM actinomycin D, 100 μM MG132, 1.56 μM methotrexate, 50 μM roscovitine, 50 μM 5,6-dichloro-1-beta-D-ribofuranosylbenzimidazole (DRB) or 7 μg/ml cycloheximide. These drugs were chosen because they have been tested previously for their effect on nucleolar proteins [28]. Controls were incubated with water (reference for cycloheximide) or DMSO (all other compounds). Nonspecific binding was negligible for cells incubated without primary and/or secondary antibodies (not shown). Following acquisition, images were corrected for background and bright holes were detected as described for Method 5. Background correction was carried out by automatically subtracting pixel intensities of an image that did not contain any cells. This image was obtained after the incubation with primary and secondary antibodies (see Figure 10a). For all HTS results, three independent experiments were carried out on three different days. At least two replicates were present on each 96-well plate, and images were acquired from 16 to 25 different sites for every sample in each of the three experiments.

### Software and basic operations

All images were analyzed with MetaXpress software (MDS Analytical Technologies). The procedures described here rely on several software operations that are used in different combinations. The selection and purpose of different filters is summarized in Figure 2. The morphology filters discussed below can be applied to 12/16 bit as well as binary images.

#### (1) Detection and demarcation of nucleoli

##### Detect dark holes

The morphology filter *Detect dark holes *identifies regions of low fluorescence intensities when nuclei are stained with DAPI (or other suitable markers). By translating black pixels to white and *vice versa*, nucleoli appear as bright objects in the nucleus. Application of the *Detect dark holes filter *on the DAPI image defines nucleoli as small objects. Using the same principle, this filter also detects nucleoli in the probe image if the fluorescent molecule of interest is more concentrated in the surrounding nucleoplasm than in nucleoli.

##### Detect light holes

The morphology filter *Detect light holes *identifies nucleoli when fluorescence is more concentrated in nucleoli than in the surrounding nucleoplasm. The nucleoli are then defined as regions of high fluorescence intensities.

##### Add function

This operation combines two images by adding their pixel intensities for pixels that are in corresponding positions. The *Add *function enhances the detection of nucleoli by increasing the difference in fluorescence intensity between the nucleolus and the surrounding nucleoplasm. To this end, cells are stained with two different nuclear markers which exclude nucleoli, and the images for both markers are then added. The resulting Add image (16 bit) serves as a source image to identify nucleoli with the *Detect dark holes filter*.

#### (2) Reduction of noise and false positives

In addition to nucleoli, other regions in the nucleus may also be recognized by the *Detect holes filter*, thereby generating false positives. Furthermore, background noise can be produced if the difference between fluorescence intensities in nucleoli and the surrounding nucleoplasm is low. This noise, if left without correction, will interfere with the proper identification of nucleoli. Two different filters, the *Median filter *and *Erode/dilate filter*, can remove such background noise, thereby limiting the number of false positives.

##### Median filter

The *Median filter *smoothes the image and removes noise by replacing each pixel intensity with the local median intensity. Specifically, after selecting the median pixel value in a specified region, the center pixel will be converted to this value. This procedure is applied to all pixels of the image; it reduces, but does not eliminate completely, noise in the processed image. To apply the *Median filter *to an image, certain parameters have to be set such as filter width, filter height and sub-sample ratio. In Additional file 1b, we provide an example of the *Median filter *setting that is appropriate for the analysis of nucleoli in HeLa, MCF7 and NIH3T3 cells. These values are determined in several trials on different images. Since the *Median filter *will not decrease pixel values for noise to 0, objects with dimensions and fluorescence intensity similar to the nucleoli may generate false positives. To prevent the analysis of false positives, the proper identification should be verified by visual inspection and incorrectly identified objects should be excluded from the analysis.

##### Erode/dilate filters

As an alternative to the *Median filter*, the combination of *Erode *and *Dilate filters *can decrease noise and false positives. The *Erode filter *brings background noise to 0, which is achieved by assigning white pixels the value 0 (black), depending on the number of black pixels in the neighborhood. Although the *Erode filter *eliminates background noise, this operation may discard some nucleoli if they are smaller or less bright than the threshold set to identify the majority of nucleoli in the cell. Although the *Erode filter *will miss some of the nucleoli, it has the advantage that the number of false positives is low. Since the *Erode filter *demarcates an area that is smaller than the authentic nucleolus, the *Dilate filter *is applied on the Erode image (see Additional file 6 for details). This operation ensures that the regions identified by the software colocalize with the actual nucleolus. The *Dilate filter *uses the same principle as the *Erode filter*, however, it assigns to black pixels the value 1 (white), depending on the number of white pixels in the neighborhood. Visual inspection is required to verify that the objects identified as nucleoli correspond to dark holes in the DAPI image.

Additional file 6 depicts a comparison between *Erode/dilate *and *Median filter *operations. The filters were applied to the same original images, and the final results are shown as overlays between the Statistical correction image (the probe image after correction for background staining) and the segments. Arrows point to the objects that are missing when the *Erode/dilate filter *operation is applied, but are identified when the *Median filter *is used. It should be emphasized that for both the *Median *and *Erode/dilate filters *it is important that settings are adjusted to optimize the proper identification of nucleoli before carrying out the analysis.

*Erode/dilate filter *functions do not require segmentation, but parameters for filter shape and diameter have to be selected. For our procedures, the filter shape was set to circle, and the optimal filter diameter was 6.

#### (3) Segmentation and quantification of fluorescence intensities in nucleoli

The median or erode/dilate filter images are analyzed with a defined set of parameters; they include size constraints and fluorescence intensity above local background. Based on this information, the software module creates segments that colocalize with nucleoli. Nucleolar fluorescence is then quantified by measuring pixel intensities in the probe image at the positions defined by segments.

##### Statistical correction (Background correction)

Prior to the measurement of pixel intensities in the nucleolus, the probe image has to be corrected for the contribution of background fluorescence. This can be achieved by the statistical correction function. Statistical correction subtracts from each pixel in the image the average intensity value of a selected region which does not contain cells. For a HTS setting a background image is automatically subtracted from all the images analyzed.

### Statistics

To measure fluorescence signals in nucleoli and nuclei, images for at least 20 cells were acquired by confocal microscopy for each of the different conditions. For HTS experiments, a minimum of 51 cells were analyzed per data point for each of the three independent experiments. For quantification, fluorescence signals were integrated over the entire nucleolus and nucleus. Results are shown as means and STDEV. Significant differences between control and stressed cells were identified with Student's *t-test *or One-way ANOVA as indicated in the figure legends.

## List of abbreviations

Act D: actinomycin D; CHX: cycloheximide; DAPI: 4', 6-Diamidino-2-phenylindole; DRB: 5,6-dichloro-1-beta-D-ribofuranosylbenzimidazole; EU: 5-ethynyluridine; Meth: methotrexate; Pol II: RNA polymerase II; Rosc: roscovitine.

## Authors' contributions

MK developed all of the protocols; MK and PB performed the experiments. MK and US wrote the manuscript. All authors read and approved the final manuscript.

## Supplementary Material

Additional file 1 **Multiple processing steps are applied to identify the nucleolar compartment**. Examples of the settings for different operations are shown. (a) If fluorescence signals in nucleoli are below the pixel intensities in the nucleoplasm, the *Detect dark holes filter *can identify nucleoli using the DAPI or probe image. (b) The *Median filter *reduces noise; regions within the yellow box were magnified 2.5-fold and contrast was increased to show changes generated by the operation. Note that all of the filters are applied only to define the nucleolar compartment; none of the operations affects the fluorescence of the probe image for which pixel values will be measured. Click here for file

Additional file 2 **Identification of nucleoli with Methods 1-4**. (a) Original confocal images show the distribution of the DNA marker DAPI (blue) and Pol II (red) as well as a GFP-tagged protein (green, probe image, Stat. correction). (b) Method 1 identifies nucleoli based on the DAPI image. (c) Method 2 employs the Pol II staining to delimit nucleoli. (d) Method 3 demarcates nucleoli by using the DAPI and Pol II images (*Add *image). It should be noted that reliance on the DAPI image only may result in identification of false positive that should be eliminated upon visual inspection. By contrast, some nucleoli could be missed when Pol II staining serves as the only reference. Using the *Add *image, a combination of the DAPI and Pol II staining, increases the accuracy of the identification process. This method is preferable when visual inspection is not permitted, as in experiments designed for HTS assays. (e) For Method 4 cells were incubated with antibodies against hsc70 and Cy3-labeled secondary antibodies (red), DNA was stained with DAPI (blue). Nucleoli with pixel intensities lower than the nucleoplasm are identified with the probe image (Method 4). Dark holes that represent nucleoli in the probe image are detected with the *Detect dark holes filter*. The *Median filter *will then reduce noise and improve the identification of nucleoli. None of these operations affects pixel values in the original probe image. Once the original probe image has been corrected for nonspecific background staining, the resulting Statistical correction image (Stat. correction) is used to quantify fluorescence signals. Based on the identification of nucleoli, the software measures pixel intensities for nucleolar segments (yellow) in the Statistical correction image. Click here for file

Additional file 3 **Nucleoli can be detected in heat-stressed cells**. HeLa cells were incubated for 1 h at 45.5°C, fixed and stained with antibodies against Pol II and RPA194, a subunit of RNA polymerase I. RPA194 was chosen as a marker for nucleoli, because it is less affected by heat than B23 and fibrillarin (unpublished data). Original images are shown in the top panels. Nucleoli are identified based on the Pol II staining (panels in the middle). This requires the *Detect dark holes *(Holes) and *Median filter *operations. Alternatively, nucleoli are demarcated by combining the information of DAPI and Pol II staining. To this end, the *Add *function, *Detect dark holes *and *Median filter *operations are performed. Click here for file

Additional file 4 **The effect of heat stress on EU staining**. For all experiments, HeLa cells were kept with EU for 6 hours, and EU was labeled with Alexa Fluor488 as described in the Methods section. In the top panels, cells were grown with EU at 37°C for 6 hours and fixed. Panels in the middle show cells that were first heat-shocked for 1 h at 45.5°C, then treated for 6 hours with EU at 37°C and fixed. For the bottom panels, cells were incubated with EU for 6 hours at 37°C, then transferred to medium without EU and stressed for 1 h at 45.5°C. Following heat shock, samples were immediately fixed and processed to visualize EU incorporation. Size bar is 20 μm. Click here for file

Additional file 5 **Quantification of nucleolar fluorescence with the *multi wavelength cell scoring module***. Details are shown for a protocol that identifies nucleoli with the DAPI image and measures florescence intensities in the probe image. (a) The flowchart provides an overview over the operations that are applied to the DAPI and probe images before nucleolar fluorescence is measured with the *multi wavelength cell scoring module*. The nucleolar compartment is delimited by applying first the *Detect dark holes *and subsequently the *Median filter*. The probe image is corrected for nonspecific background fluorescence; this generates the statistical correction image for which nucleolar fluorescence will be quantified. (b) Individual steps of the analysis are compiled into a journal which will automatically carry out all of the different operations and save data in an Excel sheet. (c) In order to run the analysis with the *multi wavelength cell scoring module*, several parameters are required to define nucleoli and quantify fluorescence signals. The parameters include size constraints for the compartment and fluorescence intensities above background for both the DAPI image (left panel) and probe image (right panel). Based on this information, the software generates segments that colocalize with nucleoli. Segments are overlaid with the statistical correction image, and fluorescence intensities are measured in the statistical correction image for the areas defined by the segmentation. Click here for file

Additional file 6 **Comparison of the *Erode/dilate *and *Median filter *function to define nucleoli with the DAPI image**. (a) Original DAPI and probe images on which the analysis was carried out. (b) Nucleoli are identified with the *Detect dark holes filter *as in Figure 3, and noise is reduced with *Erode *and *Dilate filters*. Segments generated for the nucleolar compartment are overlaid with the Dilate, DAPI or probe image, which was corrected for background fluorescence (Statistical correction). (c) For comparison, the holes image is processed with the *Median filter *to reduce noise. Note that with the *Erode/dilate *operation there is a smaller number of false positives. However, as compared to the *Median filter *operation, some of the nucleoli will be missed (white arrows). Click here for file
