# Supplementary figures and images for: Computer-based fluorescence quantification: a novel approach to study nucleolar biology
Source: BMC Cell Biol. 2011 Jun 3;12:25. doi: 10.1186/1471-2121-12-25 (PMC3126779; doi:10.1186/1471-2121-12-25)

**a** Detect dark holes

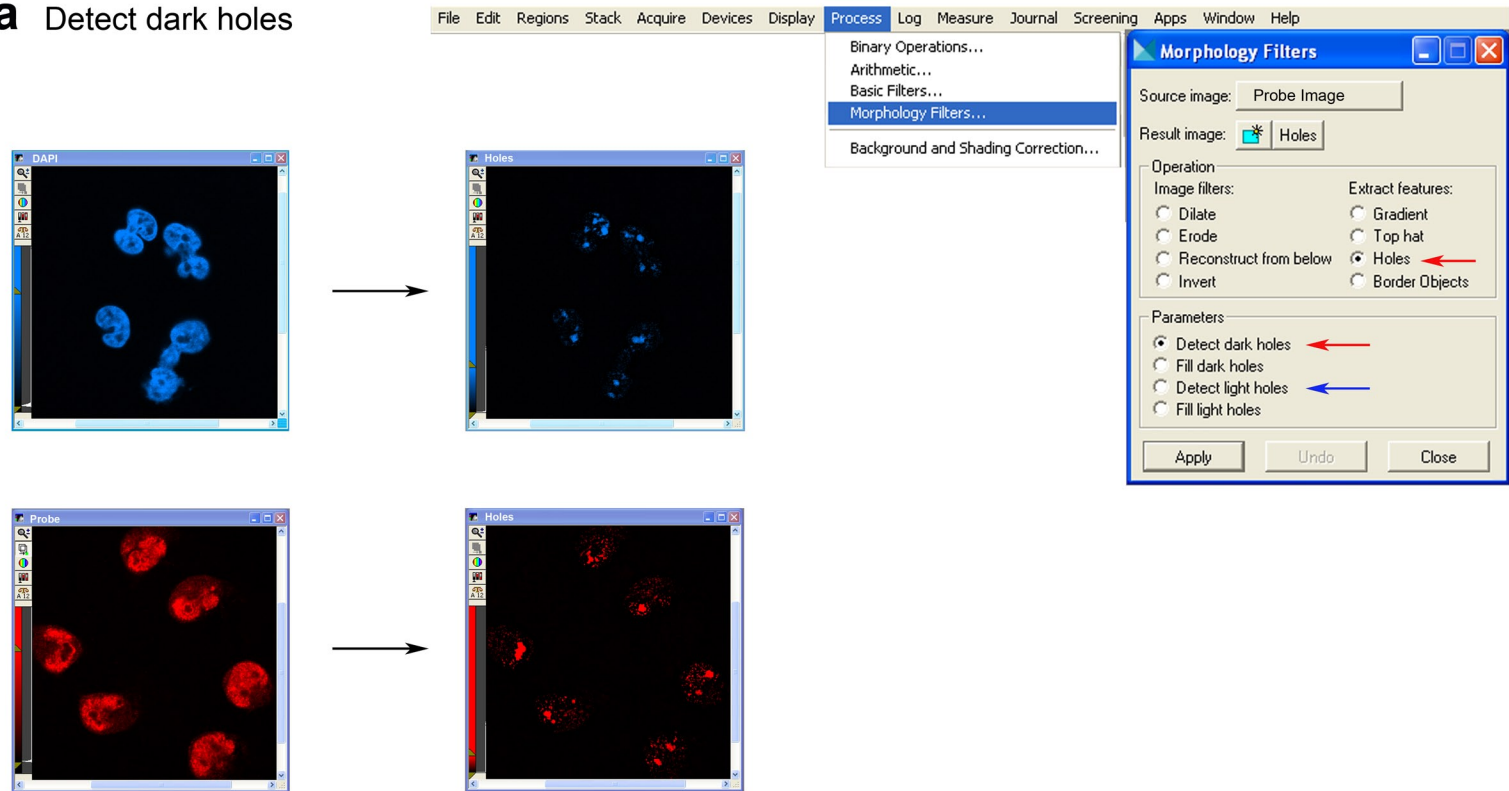

**b** Median filter

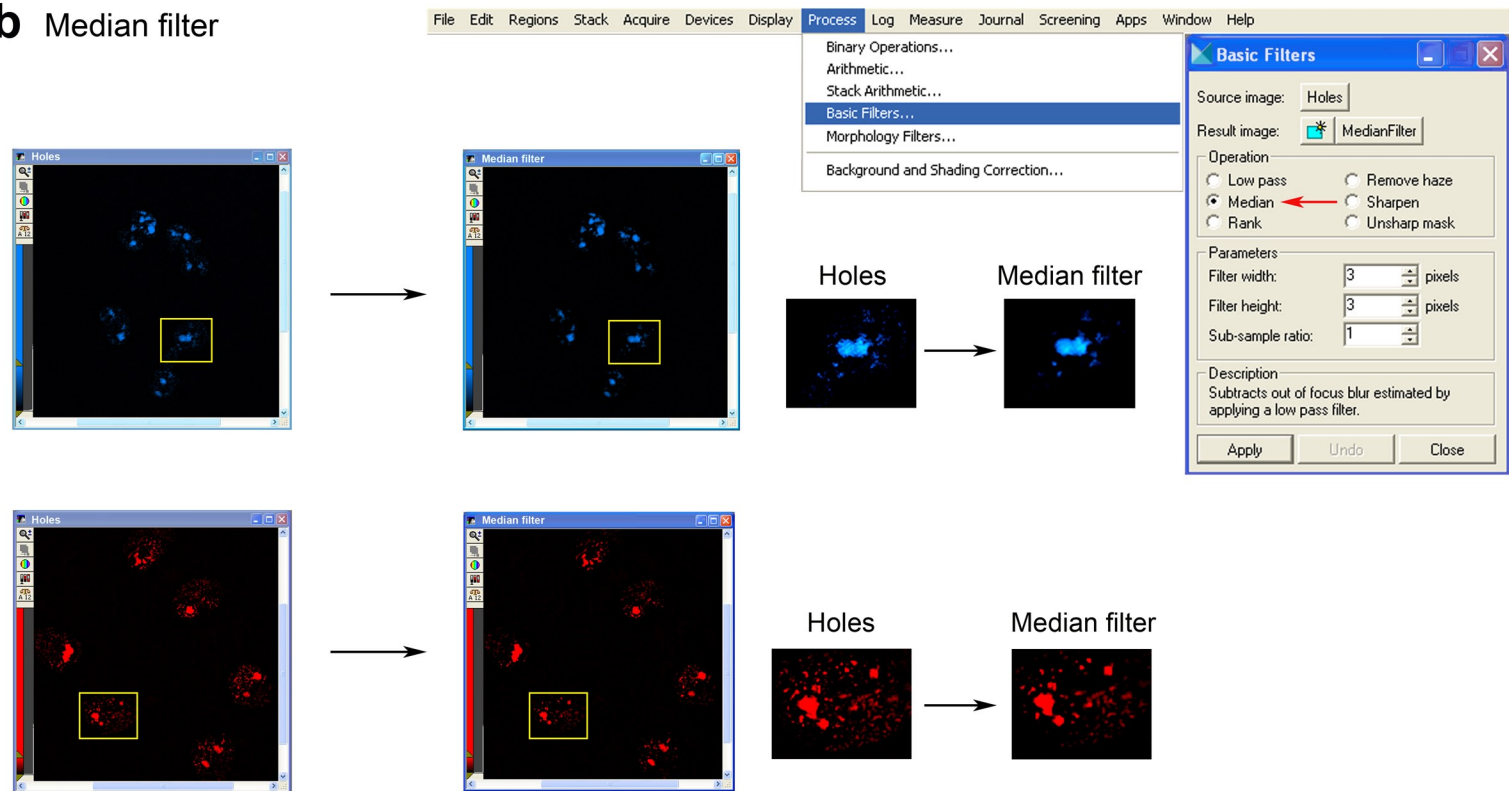

Supplement: Additional file 1 — Multiple processing steps are applied to identify the nucleolar compartment. Examples of the settings for different operations are shown. (a) If fluorescence signals in nucleoli are below the pixel intensities in the nucleoplasm, the Detect dark holes filter can identify nucleoli using the DAPI or probe image. (b) The Median filter reduces noise; regions within the yellow box were magnified 2.5-fold and contrast was increased to show changes generated by the operation. Note that all of the filters are applied only to define the nucleolar compartment; none of the operations affects the fluorescence of the probe image for which pixel values will be measured. [file 1471-2121-12-25-S1.PDF]

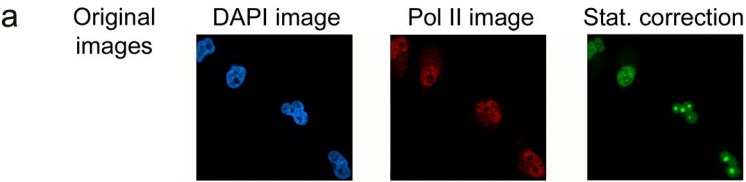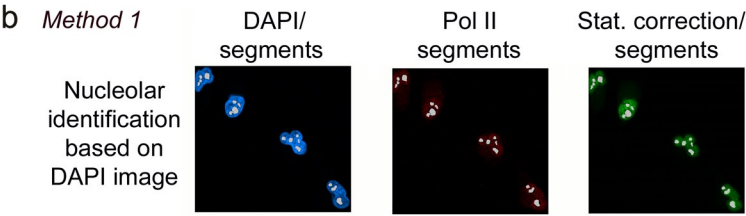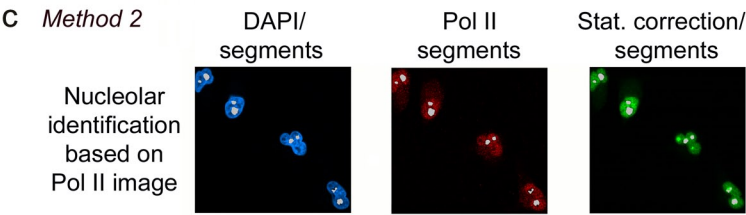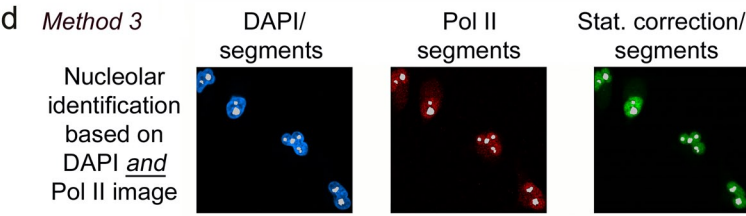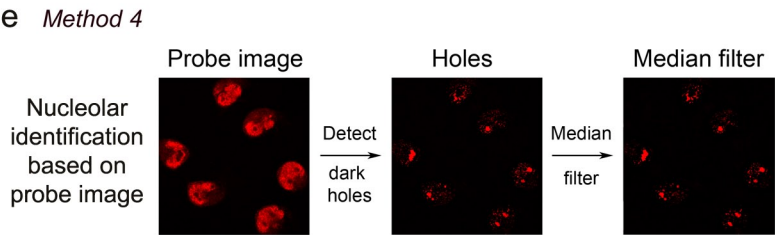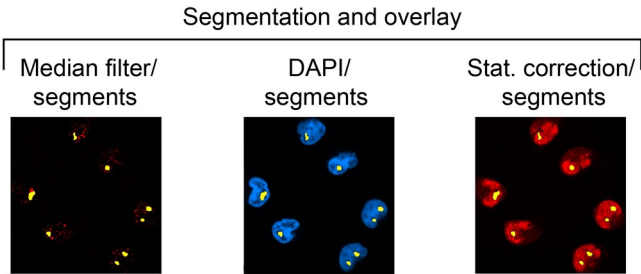

Supplement: Additional file 2 — Identification of nucleoli with Methods 1-4. (a) Original confocal images show the distribution of the DNA marker DAPI (blue) and Pol II (red) as well as a GFP-tagged protein (green, probe image, Stat. correction). (b) Method 1 identifies nucleoli based on the DAPI image. (c) Method 2 employs the Pol II staining to delimit nucleoli. (d) Method 3 demarcates nucleoli by using the DAPI and Pol II images (Add image). It should be noted that reliance on the DAPI image only may result in identification of false positive that should be eliminated upon visual inspection. By contrast, some nucleoli could be missed when Pol II staining serves as the only reference. Using the Add image, a combination of the DAPI and Pol II staining, increases the accuracy of the identification process. This method is preferable when visual inspection is not permitted, as in experiments designed for HTS assays. (e) For Method 4 cells were incubated with antibodies against hsc70 and Cy3-labeled secondary antibodies (red), DNA was stained with DAPI (blue). Nucleoli with pixel intensities lower than the nucleoplasm are identified with the probe image (Method 4). Dark holes that represent nucleoli in the probe image are detected with the Detect dark holes filter. The Median filter will then reduce noise and improve the identification of nucleoli. None of these operations affects pixel values in the original probe image. Once the original probe image has been corrected for nonspecific background staining, the resulting Statistical correction image (Stat. correction) is used to quantify fluorescence signals. Based on the identification of nucleoli, the software measures pixel intensities for nucleolar segments (yellow) in the Statistical correction image. [file 1471-2121-12-25-S2.PDF]

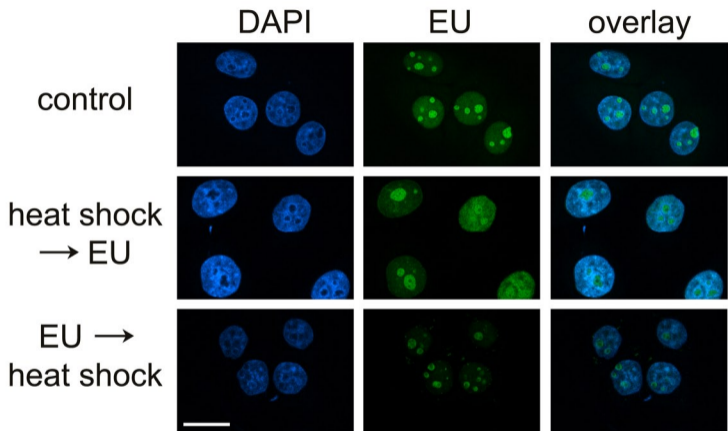

Supplement: Additional file 4 — The effect of heat stress on EU staining. For all experiments, HeLa cells were kept with EU for 6 hours, and EU was labeled with Alexa Fluor488 as described in the Methods section. In the top panels, cells were grown with EU at 37°C for 6 hours and fixed. Panels in the middle show cells that were first heat-shocked for 1 h at 45.5°C, then treated for 6 hours with EU at 37°C and fixed. For the bottom panels, cells were incubated with EU for 6 hours at 37°C, then transferred to medium without EU and stressed for 1 h at 45.5°C. Following heat shock, samples were immediately fixed and processed to visualize EU incorporation. Size bar is 20 μm. [file 1471-2121-12-25-S4.PDF]
