# Supplementary material for: Computer-based fluorescence quantification: a novel approach to study nucleolar biology
Source: BMC Cell Biol. 2011 Jun 3;12:25. doi: 10.1186/1471-2121-12-25 (PMC3126779; doi:10.1186/1471-2121-12-25)

Additional file 3

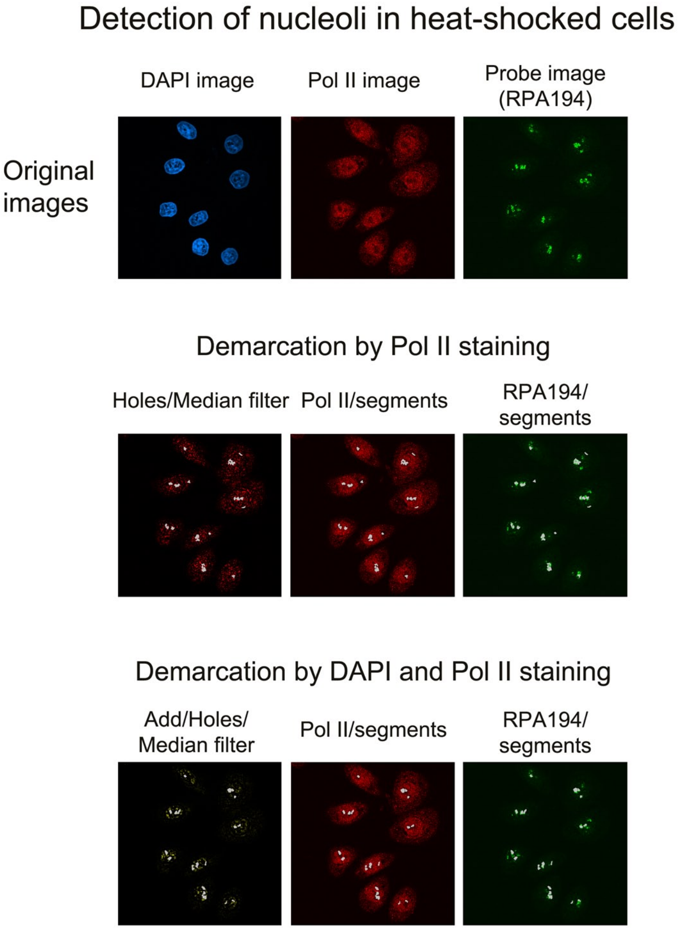

Supplement: Additional file 3 — Nucleoli can be detected in heat-stressed cells. HeLa cells were incubated for 1 h at 45.5°C, fixed and stained with antibodies against Pol II and RPA194, a subunit of RNA polymerase I. RPA194 was chosen as a marker for nucleoli, because it is less affected by heat than B23 and fibrillarin (unpublished data). Original images are shown in the top panels. Nucleoli are identified based on the Pol II staining (panels in the middle). This requires the Detect dark holes (Holes) and Median filter operations. Alternatively, nucleoli are demarcated by combining the information of DAPI and Pol II staining. To this end, the Add function, Detect dark holes and Median filter operations are performed. [file 1471-2121-12-25-S3.PDF]
