# Supplementary material for: Computer-based fluorescence quantification: a novel approach to study nucleolar biology
Source: BMC Cell Biol. 2011 Jun 3;12:25. doi: 10.1186/1471-2121-12-25 (PMC3126779; doi:10.1186/1471-2121-12-25)

Additional file 5

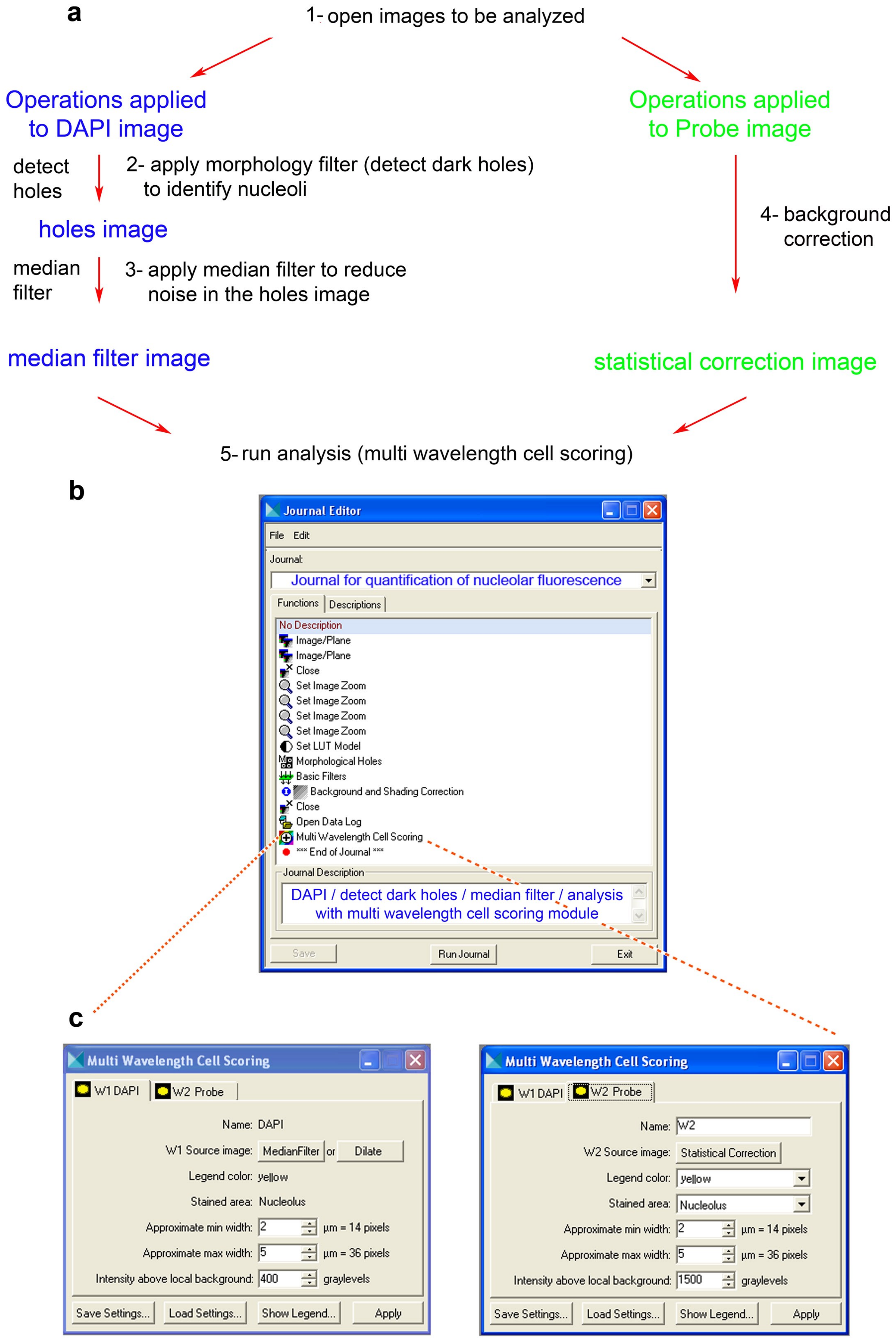

Supplement: Additional file 5 — Quantification of nucleolar fluorescence with the multi wavelength cell scoring module. Details are shown for a protocol that identifies nucleoli with the DAPI image and measures florescence intensities in the probe image. (a) The flowchart provides an overview over the operations that are applied to the DAPI and probe images before nucleolar fluorescence is measured with the multi wavelength cell scoring module. The nucleolar compartment is delimited by applying first the Detect dark holes and subsequently the Median filter. The probe image is corrected for nonspecific background fluorescence; this generates the statistical correction image for which nucleolar fluorescence will be quantified. (b) Individual steps of the analysis are compiled into a journal which will automatically carry out all of the different operations and save data in an Excel sheet. (c) In order to run the analysis with the multi wavelength cell scoring module, several parameters are required to define nucleoli and quantify fluorescence signals. The parameters include size constraints for the compartment and fluorescence intensities above background for both the DAPI image (left panel) and probe image (right panel). Based on this information, the software generates segments that colocalize with nucleoli. Segments are overlaid with the statistical correction image, and fluorescence intensities are measured in the statistical correction image for the areas defined by the segmentation. [file 1471-2121-12-25-S5.PDF]
