# Supplementary material for: Computer-based fluorescence quantification: a novel approach to study nucleolar biology
Source: BMC Cell Biol. 2011 Jun 3;12:25. doi: 10.1186/1471-2121-12-25 (PMC3126779; doi:10.1186/1471-2121-12-25)

Additional file 6

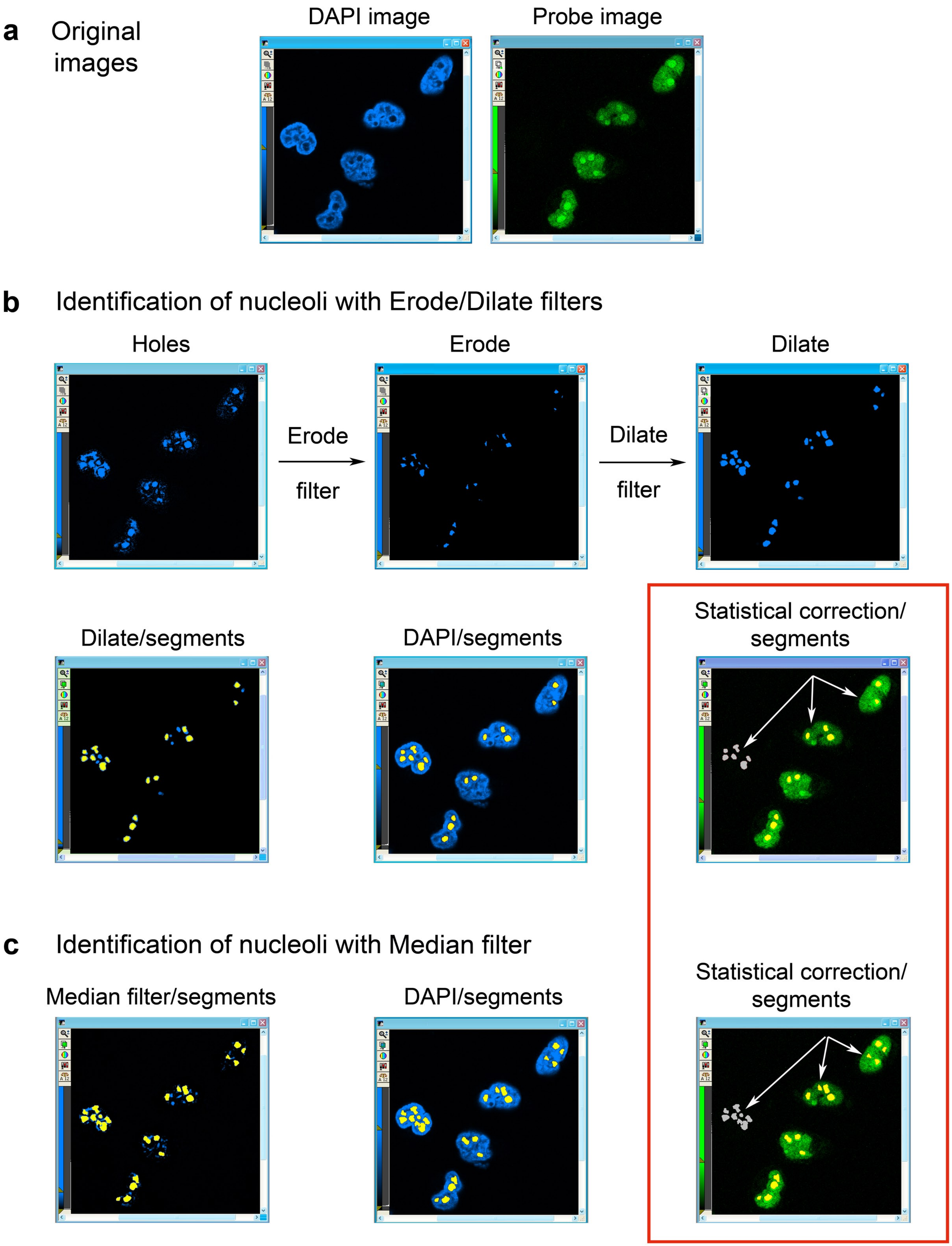

Supplement: Additional file 6 — Comparison of the Erode/dilate and Median filter function to define nucleoli with the DAPI image. (a) Original DAPI and probe images on which the analysis was carried out. (b) Nucleoli are identified with the Detect dark holes filter as in Figure 3, and noise is reduced with Erode and Dilate filters. Segments generated for the nucleolar compartment are overlaid with the Dilate, DAPI or probe image, which was corrected for background fluorescence (Statistical correction). (c) For comparison, the holes image is processed with the Median filter to reduce noise. Note that with the Erode/dilate operation there is a smaller number of false positives. However, as compared to the Median filter operation, some of the nucleoli will be missed (white arrows). [file 1471-2121-12-25-S6.PDF]
